# Identification of Genipin as a Potential Treatment for Type 2 Diabetes

**DOI:** 10.3390/ijms24032131

**Published:** 2023-01-21

**Authors:** Yajun Wu, Yao Wang, Dongmin Liu

**Affiliations:** Department of Human Nutrition, Foods, and Exercise, College of Agricultural and Life Sciences, Virginia Tech, Blacksburg, VA 24061, USA

**Keywords:** genipin, type 2 diabetes, GLP-1, L-cells, insulin resistance, lipid accumulation, mice

## Abstract

The prevalence of type 2 diabetes (T2D) has been rising dramatically in many countries around the world. The main signatures of T2D are insulin resistance and dysfunction of β-cells. While there are several pharmaceutical therapies for T2D, no effective treatment is available for reversing the functional decline of pancreatic β-cells in T2D patients. It has been well recognized that glucagon-like peptide-1 (GLP-1), which is an incretin hormone secreted from intestinal L-cells, plays a vital role in regulating glycemic homeostasis via potentiating glucose-stimulated insulin secretion and promoting β-cell function. We found that genipin, a natural compound from Elli, can directly target intestinal L-cells, leading to the secretion of GLP-1. Incubation of the cells with genipin elicited a rapid increase in intracellular Ca^2+^. Inhibition of PLC ablated genipin-stimulated Ca^2+^ increase and GLP-1 secretion, suggesting that genipin-induced GLP-1 release from cells is dependent on the PLC/Ca^2+^ pathway. In vivo, acute administration of genipin stimulated GLP-1 secretion in mice. Chronically, treatment with genipin via oral gavage at 50 mg/kg/day for 6 weeks reversed hyperglycemia and insulin resistance in high-fat-diet (HFD)-induced obese mice. Moreover, genipin alleviated the impaired lipid metabolism and decreased lipid accumulation in the liver of obese mice. These results suggest that naturally occurring genipin might potentially be a novel agent for the treatment of T2D and diet-induced fatty liver disease.

## 1. Introduction

Diabetes is still a global health issue worldwide, and the population of diabetic patients has steadily increased in recent years. More than 90% of diabetic cases are type 2 diabetes (T2D) [1]. It is well recognized that a group of intestinal hormones can increase insulin secretion in response to ingested nutrients, thus exerting an incretin effect [2]. There are two major incretin hormones, glucagon-like peptide-1 (GLP-1) and glucose-dependent insulinotropic peptide (GIP), of which GLP-1 plays a more critical role in maintaining glycemic homeostasis [3,4,5]. GLP-1 is primarily secreted by L-cells, which are enteroendocrine cells primarily located in the ileum and colon [6]. In addition to augmenting glucose-stimulated insulin secretion (GSIS) while suppressing the secretion of glucagon from pancreatic α-cells [7,8], GLP-1 was also shown to promote the survival and regeneration of pancreatic β-cells in rodent models [9]. Further, GLP-1 analogs delay stomach emptying, induce satiety, and reduce body weight gain in rodent models of obesity [10]. In patients with T2D, postprandial GLP-1 response is impaired [11], which may contribute to developing glucose intolerance and hyperglycemia. Therefore, inducing intestinal GLP-1 secretion or activating the GLP-1 signaling system can be an effective strategy for the treatment of T2D. 

Genipin is a small molecule in the fruits of *Gardenia jasminoides* Elli and *genipa americana*, and it can also be generated from an iridoid glycoside geniposide by the intestinal enzyme β-glucosidase [12,13]. Geniposide has been used to treat cancer, inflammation, metabolic diseases, and diabetes in traditional Chinese medicine for hundreds of years, although the underlying mechanism is poorly understood [14,15,16]. Interestingly, recent studies demonstrated that genipin is an inhibitor of uncoupling protein-2 (UCP-2), a mitochondrial inner membrane carrier protein expressed in various tissues [16]. It was found that the inhibition of UCP-2 by genipin in pancreatic islets increases ATP production, leading to the closure of K(ATP) channels and a subsequent increase in insulin secretion [17,18]. Given that UCP-2 may also participate in regulating GLP-1 secretion [19], our study aimed to investigate the efficacy of genipin in inducing GLP-1 secretion and its anti-diabetic potential in high-fat-diet (HFD)-induced obese, glucose-intolerant mice.

## 2. Results

### 2.1. Genipin Increased GLP-1 Secretion from L-Cells

**Figure 1:**
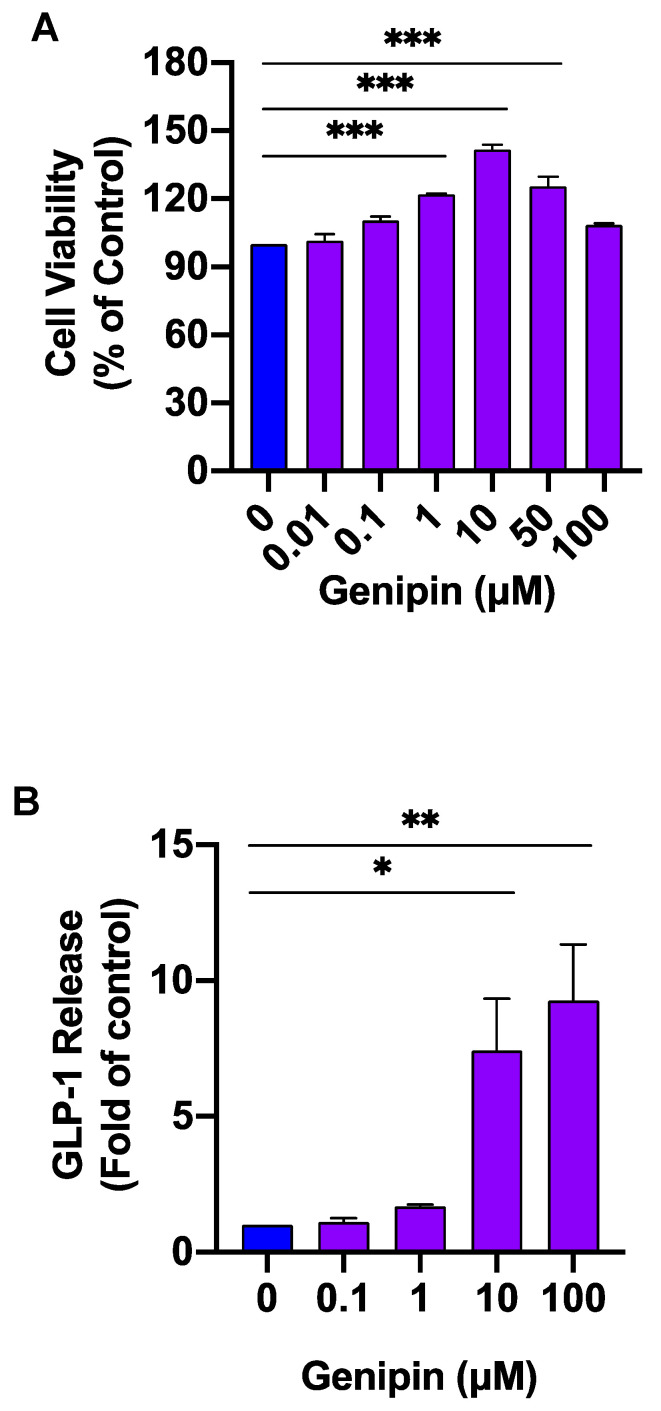
Genipin induced the secretion of GLP-1 in GLUTag L cells. GLUTag cells were treated with different concentrations of genipin for 1 h. (**A**) Supernatants were collected for GLP-1 measurement using an ELISA kit. (**B**) Cell viability was evaluated using a CellTiter-Blue^®^ cell viability assay kit. Experiments were repeated 3 times, each with duplicate determinations (*n* = 4). Data are presented as means ± SEM. * *p* < 0.05, ** *p* < 0.01 and *** *p* < 0.001 vs. control.

We investigated whether genipin can directly target L-cells to promote GLP-1 secretion. In that regard, GLUTag L-cells were treated with various concentrations of genipin for 1 h. As shown in Figure 1A, genipin was potent in stimulating GLP-1 secretion from L-cells, with 10 μM and 100 μM concentrations increasing GLP-1 release approximately 5-fold over the control. Exposure of the cells to genipin from 0.01–100 μM did not reduce cell survival, suggesting that genipin-induced hormone release is not due to its potential cytotoxicity.

### 2.2. Genipin-Induced GLP-1 Secretion Was Mediated via PLC/Ca^2+^ Signaling

**Figure 2:**
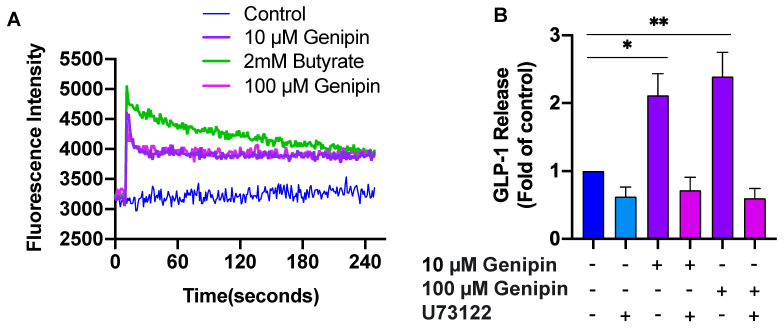
Genipin-induced GLP-1 release was mediated via the PLC/Ca^2+^ signaling pathway. (**A**) Suspended GLUTag cells were pretreated with Fluo-4AM and then incubated with genipin (10 µM, 100 µM), butyrate (2 mM), or control. The [Ca2+]_i_ response was measured using a fluorescence plate reader. A representative image from 3 independent experiments is shown. (**B**) GLUTag cells were pretreated with 10 µM U73122 for 30 min, followed by the addition of genipin (10 µM, 100 µM) or vehicle for another 1 h. Supernatants were collected for GLP-1 measurement. Data are presented as means ± SEM (n = 3). * *p* < 0.05 and ** *p* < 0.01 vs. control.

We then addressed the mechanisms by which genipin induces GLP-1 production from L-cells. We first examined intracellular [Ca^2+^]_i_ levels, as rapid increases in intracellular Ca^2+^ are critical for triggering GLP-1 secretion [20]. To this end, we preincubated GLUTag cells with Fluo-4AM followed by adding 10 μM or 100 μM genipin or 2mM butyrate (positive control). The result showed that the cells exposed to genipin induced a rapid increase in [Ca^2+^]_i_ (Figure 2A), although the magnitude was lower than that elicited by 2 mM butyrate. To determine whether genipin-induced GLP-1 secretion was phospholipase C (PLC)-dependent, GLUTag cells were pre-treated with U73122, a specific antagonist of PLC [21]. After 30 min, genipin-induced GLP-1 secretion was measured. As shown in Figure 2B, a blockage of PLC abolished genipin-induced GLP-1 secretion from L-cells, indicating that genipin-induced GLP-1 release was mediated via the PLC/Ca^2+^ signaling pathway.

### 2.3. Acute Administration of Genipin Increased Circulating GLP-1 Levels in Mice 

**Figure 3:**
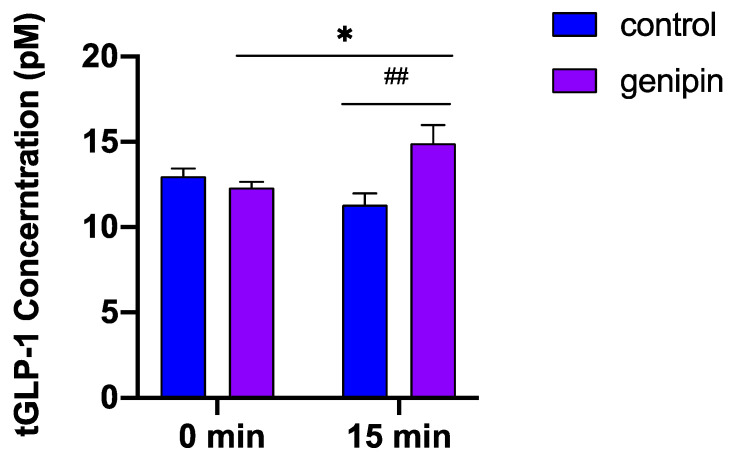
Acute administration of genipin increased plasma GLP-1 in vivo. Mice were orally administered 50 mg/kg genipin or vehicle. Blood samples were drawn before treatment (0 min) or 15 min after gavage, with total GLP-1 levels in plasma measured using an ELISA kit. The experiment was repeated twice at a 1-week interval. Data are shown as means ± SEM (n = 11 mice/group). * *p* < 0.05 vs. 0 min and ## *p* < 0.01 vs. control.

Since genipin induced GLP-1 secretion in L-cells, we next tested whether it could stimulate GLP-1 secretion in vivo. Mice were fasted for 5 h, and then blood was drawn before and 15 min after administering genipin (50 mg/kg) or vehicle (2% methylcellulose) via oral gavage. There were no significant differences in basal GLP-1 concentrations between the genipin and control groups (13.0 ± 1.6 pM vs. 12.3 ± 1.2 pM), but acute administration of genipin significantly increased plasma GLP-1 levels compared to the control (14.4 ± 3.9 pM vs. 10.9 ± 1.7 pM) (Figure 3). This result suggests that the finding that genipin directly induces GLP-1 secretion in vitro is physiologically relevant, thus providing evidence that genipin may possess an anti-diabetic property.

### 2.4. Genipin Decreased Blood Glucose in HFD-Fed Obese Mice

**Figure 4:**
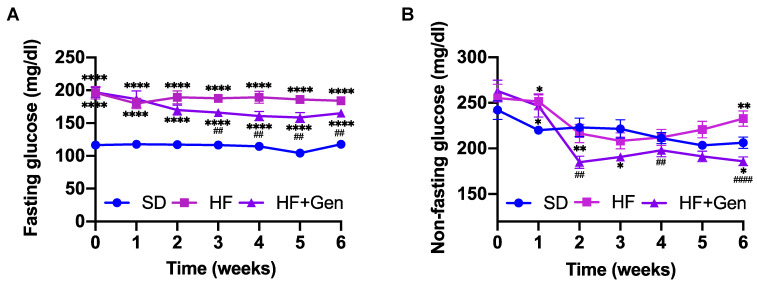
Genipin ameliorated hyperglycemia in HFD-induced obese mice. Mice were fed an HFD or SD for 10 weeks, and were then given 50 mg/kg genipin or vehicle via oral gavage. Fasting blood glucose (**A**) and non-fasting blood glucose (**B**) were measured weekly during the treatment. Data are presented as mean ± SEM (n = 12 mice for SD group, 11 mice each for HF and genipin-treated (HF + Gen) groups). * *p* < 0.05, ** *p* < 0.01 and **** *p* < 0.0001 vs. SD mice; ## *p* < 0.01, #### *p* < 0.0001 vs. HF mice.

Next, we conducted an animal study to investigate whether genipin has the therapeutic potential to treat diabetes in a murine model with obesity and glucose intolerance. When fed HFD for 10 weeks, C57/BL/6J male mice became obese, insulin resistant, and glucose intolerant compared with SD-fed mice (data not shown). The diet-induced obese mice were then orally administered 50 mg/kg genipin or vehicle once daily for 6 weeks. As shown in Figure 4A, genipin treatment significantly lowered fasting blood glucose after 3 weeks of treatment compared with the obese control group. Non-fasting blood glucose levels in mice treated with genipin were also significantly lower at the end of 2 weeks of treatment, but this difference disappeared at the end of 3 weeks of treatment (Figure 4B).

### 2.5. Genipin Improved Insulin Resistance in HFD-Induced Obese Mice

**Figure 5:**
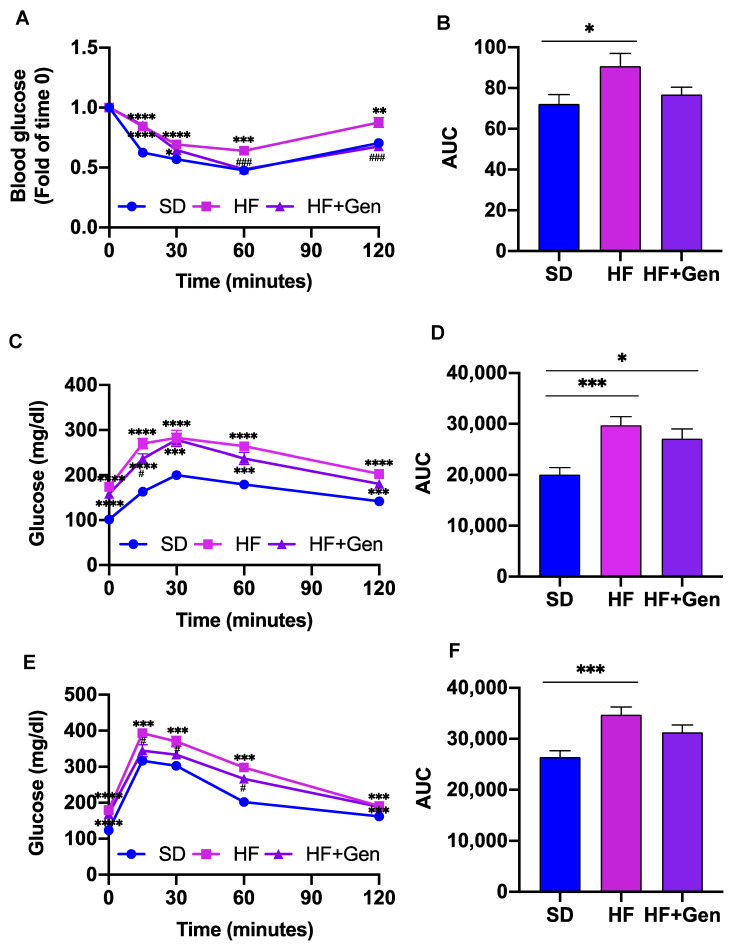
Genipin improved insulin resistance and glucose tolerance in HFD-induced obese mice. ITT (**A**) was performed after 4 weeks of treatment. Blood glucose was normalized to the 0 min of each group. PTT (**C**) and GTT(**E**) were performed after 5 weeks and 6 weeks of treatment, respectively. The corresponding area under the curve (AUC) for these tests is presented in (**B**,**D**,**F**). Data are presented as mean ± SEM (n = 12 mice for SD, 11 mice each for HF and genipin-treated (HF + Gen) groups). * *p* < 0.05, ** *p* < 0.01, *** *p* < 0.001, **** *p* < 0.0001 vs. SD mice; # *p* < 0.05 and ### *p* < 0.001 vs. HF mice.

Since genipin alleviated hyperglycemia, we next assessed whether it could promote insulin sensitivity and reverse glucose dysregulation in obese mice. In this regard, we performed an insulin tolerance test (ITT), pyruvate tolerance test (PTT), and glucose tolerance test (GTT) at the 4th, 5th, and 6th weeks, respectively. As shown in Figure 5, obese mice displayed an impairment of insulin sensitivity and pyruvate and glucose tolerance. Genipin treatment significantly improved insulin resistance (Figure 5A,B) and glucose tolerance (Figure 5E,F), whereas it had no significant effect on pyruvate tolerance (Figure 5C,D), suggesting that genipin may not modulate hepatic gluconeogenesis.

### 2.6. Genipin Did Not Alter Body Weight and Food Intake in Obese Mice

**Figure 6:**
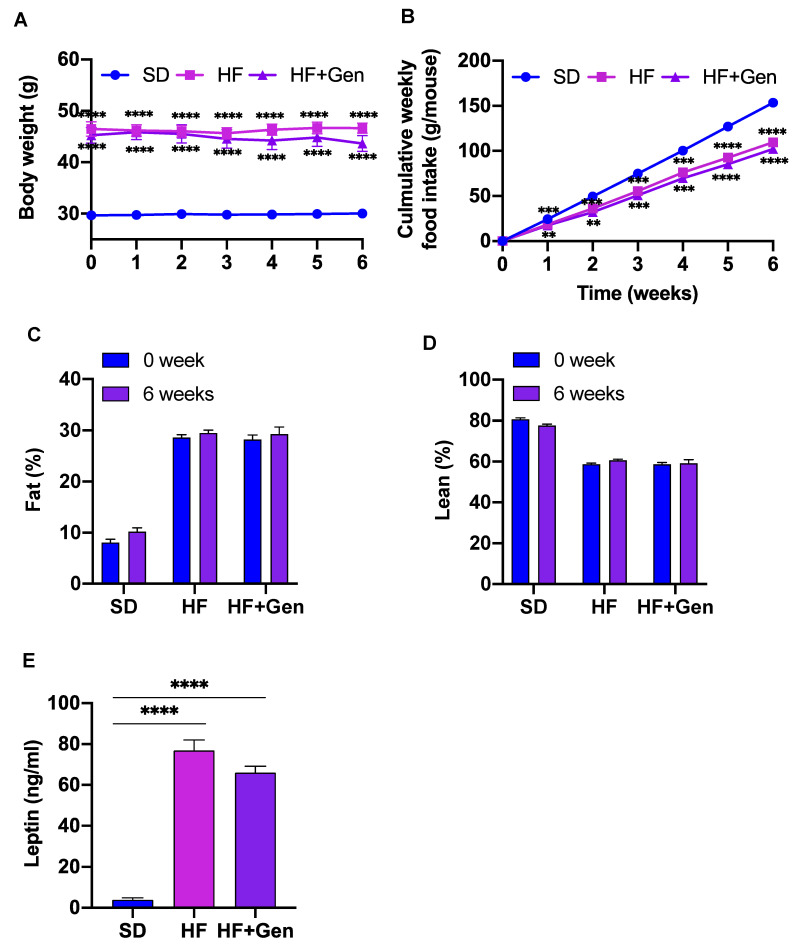
Genipin did not alter BW gain and food intake in obese mice. BW (**A**) and food intake (**B**) were recorded weekly. Body composition (**C**,**D**) was measured before and after the treatment. Blood samples were collected after treatment and plasma leptin (**E**) was determined using a commercial kit. Data are presented as mean ± SE or SEM. ** *p* < 0.01, *** *p* < 0.001 and **** *p* < 0.0001 vs. SD.

Body weight (BA) and food intake were measured every week during treatment with genipin. Genipin moderately reduced BW compared with the baseline BW (43.64 ± 5.0 g vs. 45.23 ± 5.11 g), but this change did not reach statistical significance (Figure 6A). In addition, daily food intake between the HFD group and genipin-treated mice did not significantly differ (Figure 6B). Further, the body composition of genipin-treated and untreated obese mice was similar (Figure 6C,D). Consistently, genipin treatment had no effect on plasma leptin (Figure 6E), a hormone acting in the brain to regulate appetite and fat storage [22]. Hence, genipin regulation of glucose homeostasis was not due to reducing appetite and BW.

### 2.7. Genipin Improved Lipid Profile in HFD-Induced Obese Mice

**Figure 7:**
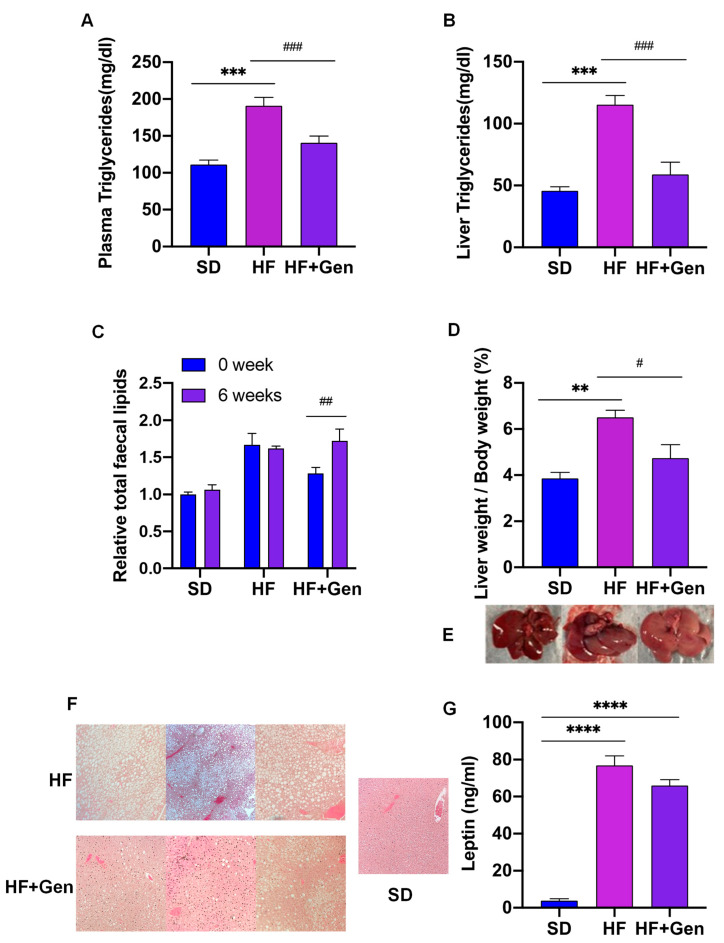
Genipin alleviated HFD-induced lipid accumulation and dysfunction of the livers in obese mice. (**A**,**B**) At the end of the 6-week treatment, mice were euthanized and blood and liver samples were collected for measuring triglyceride. (**C**) Lipid content in feces collected at 1 week and 6 weeks of treatments were measured. (**D**) Livers were harvested for weight measurement. (**E**,**F**) A set of representative pictures for liver appearance and H&E-stained images of the liver sections with 200× magnification are provided. (**G**) ALT levels in plasma were measured using an assay kit. ** *p* < 0.01, *** *p* < 0.001, **** *p* < 0.0001 vs. SD mice; # *p* < 0.05, ## *p* < 0.01 and ### *p* < 0.001 vs. HF mice.

As expected, HFD feeding significantly increased triglyceride levels in obese mice compared with SD-fed mice. However, plasma and liver triglyceride contents in genipin-treated mice were almost restored to levels comparable to those in the lean mice (Figure 7A,B). To determine whether genipin affects lipid absorption, we collected mice feces during the first and the last weeks of treatment and measured the lipid content. The results showed that oral administration of genipin increased the triglyceride content in feces (Figure 7C), suggesting that genipin may inhibit lipid digestion and/or absorption in the intestine.

As the liver plays a critical role in lipid metabolism [23], the morphology of the liver was examined. Consistent with the increases in plasma triglyceride in HFD-fed mice, the livers of these mice were oversized, and the color was pale yellow compared to the livers of SD mice (Figure 7E). However, the livers from genipin-treated mice were similar to those from SD mice. In addition, the ratio of liver weight to BW and hepatic triglycerides in HFD mice was significantly higher than those of SD mice (*p* < 0.01), in which these patterns were reversed by genipin treatment (Figure 7D). Consistently, assessment of liver histology showed that obese mice displayed a significantly higher accumulation of liver lipid droplets relative to those from SD mice, which, however, were largely reversed by genipin treatment (Figure 7F), suggesting that genipin restored the impaired hepatic lipid metabolism caused by chronic HFD feeding. We then measured ALT activity, which is a sensitive marker for liver function [24]. Serum ALT activity in obese mice was drastically higher than that in lean mice, whereas it was significantly reduced in genipin-treated mice (Figure 7G), suggesting that genipin can protect against liver damage caused by chronic HFD feeding.

## 3. Discussion

It is well recognized that GLP-1 plays a critical role in maintaining glycemic homeostasis by augmenting GSIS and promoting pancreatic β-cell mass and survival [3,4,5,9]. GLP-1 secretion in response to ingested nutrients was found to be considerably impaired in T2D patients [25], which may contribute to the dysfunction of pancreatic β-cells and the development of T2D. Therefore, inducing intestinal GLP-1 secretion can be a novel strategy for preventing and treating T2D. Secretion of GLP-1 from intestinal L-cells is predominantly regulated by macronutrient consumption, particularly fatty acids [26,27,28], although glucose, amino acids, and dietary fiber may also induce GLP-1 release [29,30]. In addition, a variety of neuroendocrine factors such as neurotransmitters and neuropeptides released by the enteric nervous system, as well as secreted hormones from other enteroendocrine cell types, such as acetylcholine [22] and gastrin-releasing peptide [23], have been implicated in the regulation of GLP-1 secretion. However, treatment strategies based on these mechanisms have not been successfully developed to treat T2D. 

In this study, we explored the effect of genipin, a natural agent generated from the fruits of *Gardenia jasminoides*, on GLP-1 secretion and glycemic control in diet-induced insulin resistant and glucose-intolerant mouse models. The results indicated that genipin was able to increase GLP-1 secretion both in vitro and in vivo. While not statistically different (*p* = 0.3245), we noticed that the total GLP-1 concentrations in control mice trended down at 15 min after gavage with vehicle compared with its baseline concentrations. We speculate that the vehicle (2% methylcellulose) may have a mild negative effect on GLP-1 secretion, which needs to be examined in future studies. If this is the case, the effect of genipin on GLP-1 secretion in vivo could be underestimated. In diet-induced obese mice, genipin reduced hyperglycemia, improved insulin resistance, and protected again HFD-induced hepatosteatosis. Therefore, genipin may exert a GLP-1-mediated anti-diabetic effect and protect against obese-related liver damage.

To investigate the signaling pathway that mediates genipin-stimulated GLP-1 secretion, we first tested whether genipin increases intracellular [Ca^2+^]_i_, which is critical for triggering GLP-1 secretion [20]. We found that incubation of the cells with genipin induced a rapid increase in intracellular [Ca^2+^]_i_. We further examined the effect of genipin on the activity of PLC, which hydrolyzes PIP2 to the Ca^2+^-mobilizing second messenger IP3, thereby elevating intracellular [Ca^2+^]_i_ [31]. We found that genipin activation of GLP-1 secretion was ablated by inhibition of PLC activity, suggesting that PLC is the upstream of genipin-elicited Ca^2+^ signaling. While not specifically determined in the present study, we believe that rapid mobilization of intracellularly stored Ca^2+^ in L-cells exposed to genipin plays an essential role in its stimulated GLP-1 secretion, given that an increase in intracellular [Ca^2+^]_i_ is the indispensable signal in mediating GLP-1 secretion induced by various nutrients and factors [31]. Recently, genipin was identified to be an inhibitor of UCP-2, which is expressed in numerous tissues and has been implicated in the regulation of insulin–glucose homeostasis [16,17,18,32,33]. Inhibition of UCP-2 by genipin was found to reverse pancreatic islet dysfunction and increase insulin secretion [17,18]. More recently, it was demonstrated that inhibition of UCP-2 increases GLP-1 release [19], suggesting that UCP-2 is a negative regulator for GLP-1 secretion. However, it remains to be determined whether UCP-2 is involved in genipin stimulation of GLP-1 secretion from L-cells. 

As shown in our study, genipin ameliorated glucose intolerance in obese mice, which could be ascribed to genipin-induced GLP-1 secretion, thereby enhancing GSIS during glucose challenge [34]. We also showed that genipin ameliorated insulin resistance in obese mice, which is weight independent, as neither BW nor adiposity was changed by genipin treatment. Interestingly, blood glucose levels significantly differed between the HFD control and genipin group at 60 to 120 min after insulin administration, but no significant differences were observed during the early phase (0 to 30 min), suggesting that genipin may not directly sensitize insulin-induced glucose uptake by peripheral tissues. A previous study found that inhibition of UCP-2 by genipin reduced hypoglycemia-induced glucagon secretion from pancreatic α-cells, thereby slowing the rate of blood glucose recovery during an insulin tolerance test [35]. Hence, the observed effect of genipin on insulin sensitivity could be at least partially due to suppressed hypoglycemia-induced glucagon secretion from the islets, which needs further investigation. 

While GLP-1 can reduce gastric emptying, food intake, and BW [36], and it was shown that genipin decreased BW gain in both rats and mice [37,38], the results from the present study show that genipin treatment did not alter these variables in obese mice. The efficacy of genipin on obesity may vary based on treatment duration, dosage, animal models, or therapeutic techniques. Triglycerides are primarily synthesized by the liver. The accumulation of excessive triglycerides in the liver can result in hepatic insulin resistance and non-alcoholic fatty liver disease (NAFLD) [39]. Our results indicated that genipin reduced hepatic lipid accumulation and the elevated ALT activity by HFD feeding, which are more pronounced effects than its effect on glucose homeostasis, suggesting that the protective effects of this compound on liver function in obese mice should be noticed and further investigated. This beneficial effect of genipin may not relate to UCP-2 or GLP-1 [40]. Previous studies showed that genipin inhibited intracellular lipid accumulation in free fatty acid-treated HepG2 cells by improving the levels of PPARα, which plays an important role in regulating nutrient metabolism and energy homeostasis [41]. However, another study reported that inhibition of UCP-2 by genipin with a concentration of 5 μM significantly aggravated palmitate-induced lipid accumulation in HepG2 cells via upregulating oxidative stress [42]. Data from in vivo studies are more consistent. One study showed that genipin improved liver dysfunction by inhibiting TNF-α production [43]. Another study demonstrated that genipin improved lipid metabolism in the liver via the miR-142a-5p/SREBP-1c axis [38]. Thus, our data are consistent with the results from these studies. However, whether and how genipin protects against NAFLD is worth further investigation. 

## 4. Materials and Methods

### 4.1. Chemicals

Genipin was purchased from BOC Sciences (Shirley, NY, USA) with purity > 98%; U73122 was from Tocris Bioscience (Pittsburgh, PA, USA); Dulbecco’s Modified Eagle Medium (DMEM) was from Hyclone (GE Healthcare Bio-Sciences, Pittsburgh, PA, USA); fetal bovine serum (FBS) was from GenClone (Genesee Scientific, EL Cajon, CA, USA); penicillin–streptomycin was from Sigma (St. Louis, MO, USA); poly-D-lysine was from MP Biomedicals (Solon, OH, USA); trypsin-EDTA and GLP-1 ELISA kits for in vitro experiments were from MilliporeSigma (Burlington, MA, USA); Fluo-4AM was from ThermoFisher Scientific (Waltham, MA); bovine serum albumin (BSA), diprotin A, methylcellulose, IBMX, cyclic AMP ELISA kit, alanine aminotransferase (ALT) kit, and triglycerides colorimetric assay kit were from Cayman (Ann Arbor, MI, USA); leptin assay kit was from Bertin Pharma (Montigny-le-Bretonneux, France); total GLP-1 ELISA kits for plasma assay were from Crystal Chem (Elk Grove Village, IL, USA); CellTiter-Blue^®^ cell viability assay reagent was from Promega (Madison, WI, USA); glucose meter and strips were from AgaMatrix (Salem, NH, USA); and all other chemicals were from Sigma (St. Louis, MO, USA).

### 4.2. Cell Culture

Mouse GLUTag L-cells were maintained in DMEM (5.5 mM glucose) supplemented with 10% fetal bovine serum (FBS) and 1% penicillin–streptomycin [44]. Cells were maintained at 37 °C with 5% CO_2_ until reaching 80–85% confluence. The passage numbers of GLUTag L-cells in all the experiments were between 25 and 35.

### 4.3. Measurement of GLP-1 Secretion

GLUTag L-cells were trypsinized with 0.05% trypsin-EDTA and then seeded into 24-well plates coated with poly-D-lysine 24 h before the study. The following day, cells (~80% confluence) were incubated with Krebs Ringer Buffer (KRB, 129 mM NaCl, 2.5 mM CaCl2, 1.2 mM MgSO4, 4.8 mM KCl, 1.2 mM KH2PO4, 10 mM HEPES, 5 mM NaHCO3, pH 7.4) supplemented with 0.2% BSA for 30 min and then treated with genipin (0.1 µM, 1 µM, 10 µM, and 100 µM) dissolved in DMSO, or vehicle with the same concentration of DMSO for 1 h. To examine the role of PLC, cells were preincubated with 10 µM U73122 for 30 min and then treated with 10 µM or 100 µM genipin for 1 h. Supernatants were collected for measuring GLP-1 levels using a GLP-1 assay kit. Cells were then lysed with 0.1 M HCl to measure the protein content and GLP-1 levels were then normalized to the protein concentrations from the same samples.

### 4.4. Cell Viability Assay

GLUTag L-cells were seeded into 96-well tissue culture microplates with a density of 1 × 104 cells/well. After 24 h, cells were treated with different concentrations of genipin (0, 0.01 µM, 0.1 µM, 1 µM, 10 µM, 50 µM, and 100 µM) for 1 h. Cell viability was then examined using a CellTiter-Blue^®^ cell viability assay kit. Briefly, 20 µL of CellTiter-Blue^®^ reagent was added to each well and cells were incubated at 37 °C for another 1 h. Cell viability was measured by recording fluorescence at 540 nm/590 nm.

### 4.5. Measurement of Intracellular Calcium Concentrations [Ca^2+^]_i_

GLUTag L-cells were seeded into a black 96-well microplate at a density of 3.0 × 10^4^ cells/well and cultured in low-glucose DMEM supplemented with 1% FBS and 1% penicillin–streptomycin for 16 h, as previously described [45]. Cells were then washed with KRB and loaded with 2 µM Fluo-4AM in Ca^2+^-free KRB buffer at 37 °C in the dark for 1 h. The cells were washed again and incubated in Ca^2+^-free KRB buffer for 30 min at room temperature to allow fura-4AM de-esterification. For intracellular Ca^2+^ measurement, basal signals of Fluo-4 AM-loaded cells were recorded for 10 s and Ca^2+^-free KRB containing 10 µM genipin or 2 mM butyrate (positive control) was then injected. The fluorescence intensities were recorded every second for 240 acquisition cycles at 495 nm excitation and 518 nm emission using a Spectro fluorophotometer (FLUOstar OPTIMA, Cary, NC, USA). 

### 4.6. Animal Studies

C57/BL/6J male mice (8 weeks old) were purchased from The Jackson Laboratory (Bar Harbor, ME, USA). Mice were housed under 23 °C and 12 h light–dark cycle with ad libitum access to food and water. All procedures performed in animal studies were approved by the Institutional Animal Care and Use Committee at Virginia Tech.

#### 4.6.1. The Effect of Acute Administration of Genipin on GLP-1 Secretion

To determine whether genipin stimulates GLP-1 secretion in vivo, 10-week-old C57/BL6 male mice fed a standard chow diet (SD) were randomly divided into two groups (n = 6/group). After being fasted for 5 h, blood samples were collected from the tail vein into EDTA tubes (Microvette) pre-coated with 50 μM diprotin A. Mice were then given genipin (50 mg/kg) or vehicle (2% methylcellulose) via oral gavage. Blood samples were collected after 15 min, with plasma total GLP-1 concentrations measured using a mouse GLP-1 ELISA kit.

#### 4.6.2. Chronic Effects of Genipin in Obese Diabetic Mice

Male C57/BL6J mice of 8 weeks of age were either fed a SD (n = 11) or HFD (n = 22 mice) with 60% calories from fat (Research Diet Inc., New Brunswick, NJ, USA). After 10 weeks when mice fed an HFD became obese, insulin resistant, and glucose intolerant, as shown in our recent study [46], diet-induced obese mice were divided into two groups (n = 11/group) with comparable body weight (BW) and blood glucose and then administered genipin (50 mg/kg, once a day) or vehicle (2% methylcellulose) via oral gavage for 6 weeks. Age-matched healthy control mice fed SD were given a vehicle for 6 weeks. BW, food intake, fasting, and non-fasting blood glucose were measured weekly, with body composition determined by using an NMR Lean/Fat Analyzer for small animals (Bruker, Billerica, MA, USA) at the beginning and the end of the study.

#### 4.6.3. Insulin Tolerance Test (ITT), Pyruvate Tolerance Test (PTT), and Glucose Tolerance Test (GTT)

ITT, PTT, and GTT were measured at the 4th, 5th, and 6th week, respectively. For ITT, mice were fasted for 5 h followed by i.p. injection of insulin (1.5 U/kg BW). Blood was drawn at 0, 15, 30, 60, and 120 min and blood glucose levels were measured. For PTT, mice were fasted for 16 h, and then the basal blood glucose levels were measured. Afterward, mice were given pyruvate (1 g/kg BW) or vehicle via intraperitoneal (i.p.) injection. Blood samples were drawn from the tail vein at 0, 15, 30, 60, and 120 min for measuring glucose using a glucose meter. For GTT, mice were fasted for 14 h and then a bolus of glucose (1 g/kg BW) was administered via i.p. injection. Blood glucose levels were measured at 0, 15, 30, 60, and 120 min post-injection of glucose.

#### 4.6.4. Histological Analysis

At the end of the study, liver samples were collected after the euthanasia of the mice and fixed in 4% paraformaldehyde. Embedding and sectioning of liver samples were performed by AML Laboratories Inc. (Jacksonville, FL, USA). Tissue sections were de-paraffinized with xylene, rehydrated in graded ethanol solutions, and then stained with hematoxylin and eosin (H&E), as previously described [47]. 

#### 4.6.5. Fecal Lipid Extraction and Measurement

Mice feces were collected for 3 consecutive days at the beginning and the last week of treatment. Feces were weighed and dried and lipids were then extracted in a chloroform–methanol (2:1) mix solution. Suspensions were centrifuged at 1000× *g* for 10 min at room temperature. The chloroform: methanol phases were collected for lipid measurement, as described [48]. 

#### 4.6.6. Biochemical Analyses

Blood samples were collected by cardiac puncture immediately after the mice were euthanized, with blood samples transferred into EDTA pre-coated tubes and then centrifuged at 1000× *g* for 15 min at 4 °C. Plasma was collected from the supernatant and stored at −80 °C. Plasma triglycerides and leptin were measured using assay kits. Livers were harvested from control and genipin-treated mice after the blood sample collection and stored at −80 °C. Liver triglyceride was extracted and tested according to the manufacturer’s instructions.

### 4.7. Statistical Analysis 

Data were analyzed by one-way or two-way analysis of variance (ANOVA) by using GraphPad Prism 8 (GraphPad Software, La Jolla, CA, USA). A *p* < 0.05 was considered to be a significant difference. Values are presented as the standard error of the mean (SEM).

## 5. Conclusions

We found that genipin induces GLP-1 secretion from intestinal L-cells via a PLC/Ca^2+^-mediated mechanism. Treatment with genipin restored glucose homeostasis and insulin sensitivity in HFD-induced obese mice, possibly due to increased GLP-1 release in vivo. In addition, genipin effectively improved lipid metabolism and reduced liver accumulation in obese mice. Given these results, genipin may be a natural agent that can promote the endogenous GLP-1 secretory function of intestinal L-cells and protect against diet-induced liver damage to maintain glucose homeostasis. Therefore, genipin could be a promising lead compound for developing treatments for NAFLD and T2D. 

## Data Availability

The data that support the findings of this study are available from the corresponding author upon reasonable request. Some data may not be made available because of privacy or ethical restrictions.
